# Chronic clomipramine treatment increases hippocampal volume in rats exposed to chronic unpredictable mild stress

**DOI:** 10.1038/s41398-022-02006-9

**Published:** 2022-06-10

**Authors:** Shanshan Zhang, Juntao Hu, Guixue Liu, Haoran Wu, Meihui Li, Chenye Shi, Qiong Liu, Wensheng Li

**Affiliations:** 1 Department of Anatomy, Histology and Embryology, School of Basic Medical Sciences, Fudan University, Shanghai, China; 2 Key Laboratory of Medical Imaging Computing and Computer Assisted Intervention of Shanghai, Shanghai, China; 3 Department of General Surgery, Zhongshan Hospital, Fudan University, Shanghai, China; 4 State Key Laboratory of Medical Neurobiology and MOE Frontiers Center for Brain Science, and Institutes of Brain Science, Fudan University, Shanghai, 200032 China

**Keywords:** Neuroscience, Psychiatric disorders

## Abstract

It is well known that neuroinflammation is closely related to the pathophysiology of depression. Due to individual differences in clinical research, the reduction of hippocampal volume in patients with depression is still controversial. In this experiment, we studied a typical kind of tricyclic antidepressant, clomipramine. We designed a series of experiments to find its role in depressive-like behavior, hippocampal neuroinflammation as well as hippocampal volume changes induced by chronic unpredictable mild stress (CMS). Rats exhibited defective behavior and hippocampal neuroinflammation after 12 weeks of CMS, which included elevated expression of cleaved interleukin-1β (IL-1β) and NLRP3 inflammasome together with the activation of microglia. Rats exposed to CMS showed weakened behavioral defects, reduced expression of IL-18, IL-6, and IL-1β along with reversed activation of microglia after clomipramine treatment. This indicates that the antidepressant effect of clomipramine may be related to the reduced expression of NLRP3 inflammasome and cleaved IL-1β. Moreover, we found an increased hippocampal volume in rats exposed to CMS after clomipramine treatment while CMS failed to affect hippocampal volume. All these results indicate that the NLRP3 inflammasome of microglia in the hippocampus is related to the antidepressant effects of clomipramine and CMS-induced depressive-like behavior in rats.

## Introduction

It is estimated that more than 0.3 billion people worldwide suffer from major depressive disorder (MDD), which is a really frequent mental disease nowadays [1]. Risk factors causing depression vary from family history, recent life stressors to early life abuse and neglect [2]. There is no consensus on the pathogenesis of depression. Nonetheless, the neuroinflammation hypothesis has been on the rise in recent years [2].

Many studies have demonstrated that some anti-inflammatory agents can act as antidepressants [3]. Microglia participate in the onset and continuation of inflammatory response after acute injury of the central nervous system (CNS) [4] and several neurodegenerative diseases [5, 6] as the primary immune cells in the CNS. In a CMS-induced depression animal model, it was found that the activated microglia in the brain increased significantly [7]. In addition, a meta-analysis study uncovered that men with MDD showed an increase in genes related to oligodendrocytes and microglia [8]. The activated microglia initiates the activation of the nucleotide-binding domain, leucine-rich-containing family, pyrin domain containing-3 (NLRP3) inflammasome, promoting the cleavage and subsequent secretion of pro-inflammatory factors in MDD [9]. This process is related closely to the etiology of MDD [10, 11].

Currently, antidepressants mainly target the monoaminergic neurotransmitter system [12] while only 60–70% of MDD patients have a response to these treatments [13]. The classical tricyclic antidepressant clomipramine is used for MDD extensively. Our previous research demonstrated that it reverses defective behavior and inhibits hippocampal neurogenesis in chronic unpredictable stress-treated adult rats [14, 15]. Also, in an acute stress model, it was suggested that the improvement of depression-related symptoms by clomipramine treatment has something to do with NLRP3 inflammasome [16]. However, the downstream molecular mechanisms through which clomipramine improves depressive behavior have not yet been fully understood. Its influence on the NLRP3 inflammasome and immunomodulatory potential is of particular interest.

The hippocampus, a complex brain area related to depression, exhibits functional as well as morphological changes under stress [17]. It is suggested that hippocampal volume decreased under depressive status in many studies [18, 19] while some have different conclusions [20, 21]. Besides, how the hippocampal volume changes after clomipramine treatment remain ill-defined.

From there, we investigated whether chronic clomipramine treatment would reverse depressive-like behaviors induced by CMS and examined the underlying molecular mechanisms. Furthermore, we analyzed changes in the hippocampal volume of rats after CMS with or without clomipramine treatment

## Materials and methods

### Experimental design

**Fig. 1:**
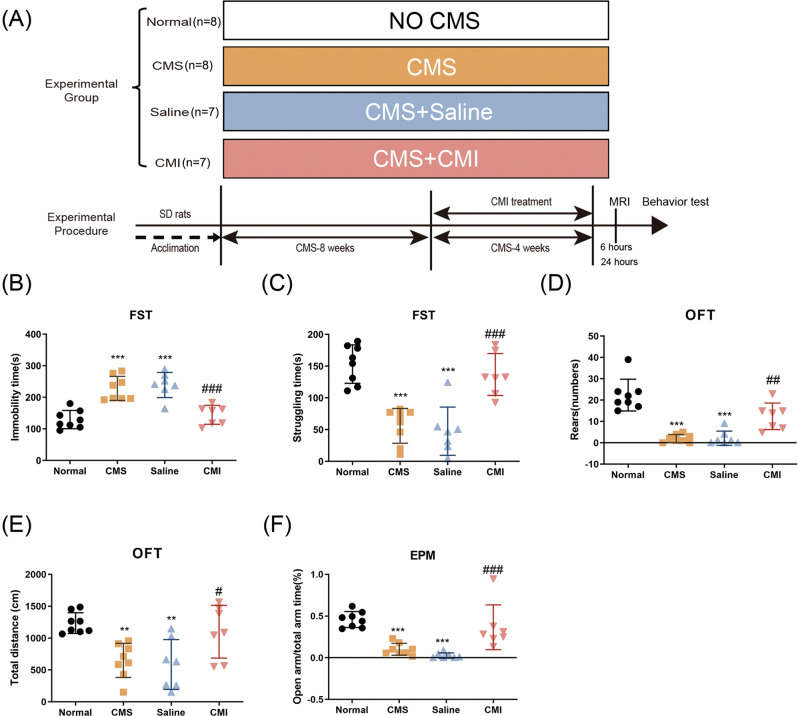
Chronic clomipramine treatment reversed CMS-induced depressive- and anxiety-like behaviors. **A** Schematic diagram of our experimental design. **B** Immobility time and **C** struggling time in the FST. **D** Rearing numbers and **E** total distance traveled in the OFT. **F** Percentage of open-arm time in the EPM test (*n* = 7–8/group). All data are expressed as the mean ± SD. #*p* < 0.05, ##*p* < 0.01, ###*p* < 0.001 compared to saline-treated rats. **p < 0.01, ***p < 0.001 compared to non-stressed control rats.

As illustrated in Fig. 1A, there were four groups in our experiment. The animals were divided randomly into four groups based on the simple randomization strategy (the random number method), namely the normal group (*n* = 8), the CMS group (*n* = 8), the saline group (*n* = 7), and the CMI group (*n* = 7). The normal group was the normal control; the remaining three groups were exposed to 12-week CMS, among which, two groups were treated simultaneously with either saline or clomipramine (5 mg/kg, diluted in saline) for 4 weeks (weeks 9–12 of CMS). Twenty-four hours after the last CMS stressor, rats of each group received behavioral tests in sequence, then some rats were used for magnetic resonance imaging (MRI) detection, and others were sacrificed. And, brain tissue was collected. For the sake of the reduction principle, we did not set up a single clomipramine group in this experiment since our previous works as well as other studies found that clomipramine has limited effects on rodents’ behaviors or other molecules in the hippocampus [11, 22].

### Animals

Male Sprague-Dawley (SD) rats weighed between 180 and 200 g were used in our experiment. The rats were housed 2–4 per cage, under a 12-h light/dark cycle, and with free access to food and water. The stress intervention began after a 1-week habituation period. We conducted this study in line with the National Institutes of Health Guide for the Care and Use of Laboratory Animals. Our protocol was approved by the Animal Ethics Committee of School of Basic Medical Sciences, Fudan University, Shanghai, China.

### CMS procedure

CMS is a rodent model of depression in which animals are exposed to a random sequence of mild stressors. Rats were subjected to nine different stressors: water deprivation (24 h), light/dark cycle reversal, food deprivation (24 h), hot environment (40 °C for 5 min), cage shake (15 min), restraint (2 h), swimming in cold water (4 °C for 5 min), radio noise in the room (12 h), and flashing lights (12 h). These stressors were performed once per day in a random order for 12 weeks.

### Behavioral testing

We conducted the open field test (OFT), the elevated plus-maze (EPM) test, and the forced swim test (FST) in sequence 24 h (1 test/day) after the last stressor of our CMS protocol (*n* = 7–8/group). All the tests were conducted by an experimenter unaware of the rats’ group information.

As for the protocol of OFT, the tested rat was placed softly in the center of a Plexiglass box (100 cm × 100 cm × 40 cm) in a brightly lit room. During a 5-min session, animals were scored for the number of rearing behaviors exhibited and the distance traveled in the box. Animal behavior was recorded and subsequently analyzed using a video-tracking system (Shanghai Mobile Datum Information Technology Company, Shanghai, China).

The maze of EPM was shaped just like a plus sign. It consisted of three parts: two open arms (without walls on the sides of the arms, 30 cm × 5 cm), a central platform (5 cm × 5 cm), and two closed arms (with walls on the sides of the arms, 30 cm × 5 cm). The plus-maze was 50 cm above the floor and illuminated by a dim light. Individual trials lasted 5 min each and were recorded and analyzed with a video-tracking system (Shanghai Mobile Datum Information Technology Company, Shanghai, China). Open-arm time percentage ([time spent in open arms] / [time spent in total arms] × 100%) was calculated as described in our previous paper [23].

There are two sessions in the FST, 24 h apart. The first session is the pre-test stage (15 min) and the second session is the test stage (5 min). Rats were individually placed in an 18- or 15-cm-diameter glass cylinder for 15 min. The cylinder was filled with 23 ± 1 °C water, at which rodents feel comfortable, to a depth of 30 cm. Twenty-four hours later, the video camera was turned on, and then placed the rat in the water-filled cylinder container for 5 min. We defined the immobility status as no extra movement except for that required to keep its head out of the water, and the struggling status as fierce movement breaking the water with its forepaws. The results were expressed as the time (in seconds) that the tested rat stays still or struggles for 5 min.

### Immunohistochemical analysis

After the behavioral tests were completed, all the animals were euthanized under general anesthesia with pentobarbital. Rats’ brains were collected and subsequently post-fixed at 4 °C in 4% paraformaldehyde (PFA) overnight, then immersed in 20% sucrose (4% PFA as solvent) followed by 30% sucrose (in 0.1 M phosphate-buffered saline [PBS]). Brain samples were cut into 30-μm-thick sections (CM1850; Leica Microsystems, Wetzlar, Germany). Brain sections were incubated in 0.01 mol/L citrate buffer (pH 6.0), followed by high-temperature antigen retrieval. Subsequently, the brain sections were blocked in 2% (w/v) bovine serum albumin (BSA, Sigma) and then exposed to the anti-Iba-1 (1:500, Ab5076, Abcam) primary antibody mixture at 4 °C overnight. Detection of the primary antibody was performed with secondary antibody (donkey anti-goat, Alexa 488 conjugated, 1:1000, A32814, Invitrogen) for 1 h in the dark. Both antibodies were diluted with 2% BSA. Then the brain sections were washed five times with PBS away from light. Immunofluorescence sections were observed with a Leica SP5 fluorescence microscope, using excitation wavelengths of 488 nm (argon, yellow-green Cy2 immunofluorescence). A CCD spot camera was used to capture images for data analysis.

### Western blot analysis

Rat hippocampal tissue was homogenized in RIPA buffer (Thermo Scientific, Waltham, MA, USA), which contained protease inhibitors (Beyotime, Jiangsu, China). The protein samples were electrophoresed on 12% Tris-glycine SDS-PAGE gels. Then the gels were transferred to PVDF membranes (0.2 or 0.45 μm), followed by blotting with antibodies against Iba-1 (1:1000, Ab5076, Abcam), NLRP3 (1:1000, #768319, R&D), Caspase-1 p20 (detects endogenous levels of pro-caspase-1 and the caspase-1 p20 subunit) (1:1000, A27351509, AdipoGen), ASC (1:200, Sc-22514-R, Santa Cruz), IL-1β (1:1000, AF-501-NA, R&D), GAPDH (1:20,000, 60004–1–1g, Proteintech), and β-actin (1:20,000, HRP-60008, Proteintech). Primary antibody incubation was performed at 4 °C overnight. Afterward, the membranes were incubated with the secondary antibody (1:10,000) at room temperature for 2 h. The signal was captured on an ImageQuant LAS4000 mini image analyzer (GE Healthcare, Buckinghamshire, UK) (*n* = 4/group). The protein expression was quantified by analyzing band levels with ImageJ software (NIH, Bethesda, MD, USA).

### Real-time PCR (qPCR)

Trizol reagent (Invitrogen) was used to isolate total RNA from the hippocampus of the rats according to the manufacturer’s instructions. Then cDNA was synthesized with a Prime Script Kit (Bio-Rad, Hercules, CA, USA). Quantitative PCR was conducted with SYBR Premix Ex Taq (Bio-Rad) and gene-specific primers. The relative expression level of messenger RNA (mRNA) was analyzed using the 2^−ΔΔCt^ method and normalized to *GAPDH* ribosomal RNA (*n* = 4–7/group). All the oligonucleotide primers used in this study were listed in Table 1.

**Table 1:** Oligonucleotide primers used for mRNA real-time PCR.

Genes	Sense primer (5’ to 3’)	Antisense primer (5’ to 3’)
IL-1β	TTCTTTGAGGCTGACAGACC	CGTCTTTCATCACACAGGAC
IL-4	GAACCAGGTCACAGAAAAAGGGA	TGGGAAGTAAAATTTGCGAAGCA
IL-6	ACTTCCAGCCAGTTGCCTTCTTG	GGTCTGTTGTGGGTGGTATCCTC
IL-10	TGCCTTCAGCAGAGTGAAG	GGGAAGAAATCGATGACAG
NLRP3	AGTGGATAGGTTTGCTGGGATA	CTGGGTGTAGCGTCTGTTGAG
caspase-1	AGTGTAGGGACAATAAATGG	GATGGACCTGACTGAAGC
ASC	GAAGAGTCTGGAGCTGTGG	AATGAGTGCTTGCCTGTG
iNOS	GCACAGAGGGCTCAAAGG	CACATCGCCACAAACATAAA
GAPDH	CCCTTCATTGACCTCAACTAC	CTTCTCCATGGTGGTGAAGAC

### Magnetic resonance imaging (MRI) acquisition

In vivo MRI studies were conducted in rats at 6 h following the end of the stress+treatment paradigm post model establishment. The animals participating in the test were anesthetized with isoflurane (1.5–2% in 20% oxygen) and then positioned in a 7.0-Tesla small animal MR scanner (Bruker Biospec 70/20 USR, Bruker, Ettlingen, Germany). Rats were kept warm by using a heating pad. Respiration rate was monitored with a Bruker Physiogard system and kept at 20–30 breaths/min. A fast spin-echo sequence (parameters: repetition time/echo time = 4300/33 ms, field of view = 325 × 20 mm, acquisition matrix = 256 × 256, 3 average, rare factor = 8, number of slices = 40, slice thickness = 0.5 mm) was used to acquire T2-weighted images.

The hippocampi were manually delineated by the software of ITK-snap [24]. ITK-SNAP provides semi-automatic segmentation to analyze selected regions and obtain their 3D models. For each MRI image, we manually delineated the border of the hippocampus based on the rat brain atlas [25] layer by layer. And their volumes were calculated and normalized respectively by the software automatically, as well as their 3D models were built (*n* = 4/group).

### Statistical analysis

All data were analyzed with SPSS 16.0 software (SPSS Inc., Chicago, IL, USA). Data collection and analysis were performed independently by two experimenters. The results are expressed as mean ± standard deviation. The one-way analysis of variance method was used to analyze data acquired in this experiment according to the factors introduced in the experimental design. Where F ratios were significant, post hoc comparisons were made using Tukey’s post hoc test. Significance levels were set at *p* < 0.05.

## Results

### Chronic clomipramine treatment ameliorates depressive- and anxiety-like behaviors induced by CMS

The SD rats ultimately express depressive-like behavior after 12-week CMS, which is in line with our previous work [26]. The rats exposed to CMS protocol showed increased immobility time, as well as decreased struggling time, in the FST (Fig. 1B, C; *p* < 0.001, *p* < 0.001, respectively); reduced rearing number (Fig. 1D; *p* < 0.001), and the total distance moved (Fig. 1E; *p* < 0.01) in the OFT, which reflected increased desperation together with decreased locomotor activity and explorative behavior. Moreover, in the EPM test, CMS made the rats less prone to stay in the open arms when compared to the control animals (Fig. 1F; *p* < 0.001), which reflected increased anxiety. Taken together, our CMS protocol induced depressive- as well as anxiety-like behavior successfully in SD rats. Clomipramine, as a typical kind of tricyclic antidepressant, is used extensively in the treatment of depression. Chronic administrating clomipramine in rats exposed to CMS significantly meliorates depressive- and anxiety-like behaviors, indicated by less immobile time together with more time struggling in the FST (Fig. 1B, C; *p* < 0.001, *p* < 0.001, respectively) and increased the time spent in the open arms of the elevated maze (Fig. 1F; *p* < 0.001) when compared to CMS exposed rats treated by saline.

### Chronic clomipramine treatment reverses CMS-induced microglial increase and elevated expression of inflammatory factors in rats’ hippocampus

**Fig. 2:**
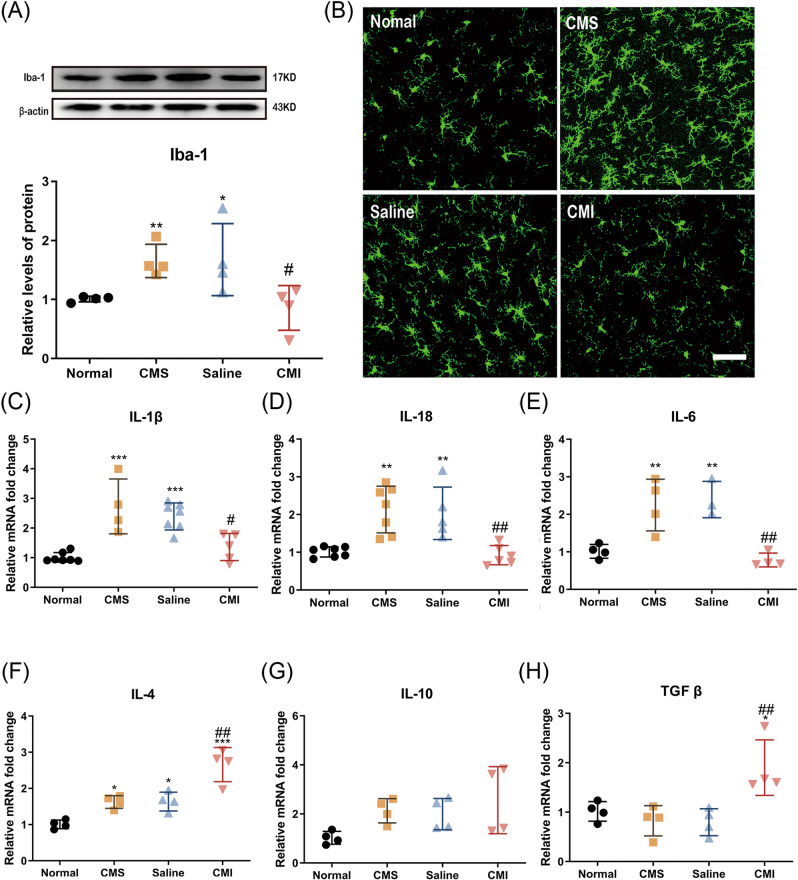
Chronic clomipramine treatment improved the activated microglia and changes in inflammation-related cytokines in rats induced by CMS. **A** The protein level of Iba-1 was analyzed by western blot (*n* = 4/group). **B** The Iba-1 staining in the dentate gyrus area of the hippocampus. Scale bar, 50 μm (*n* = 4–7/group). The mRNA level of **C** IL-1β, **D** IL-18, **E** IL-6, **F** IL-4, **G** IL-10, and **H** TGFβ was analyzed by qPCR (*n* = 4/group). All data are expressed as the mean ± SD. #*p* < 0.05, ##*p* < 0.01 compared to saline-treated rats. *p < 0.05, **p < 0.01, ***p < 0.001 compared to non-stressed control rats.

Neuroinflammation, especially in the hippocampus is considered one of the key pathological changes in MDD, and microglial cells seem to be key players in this process [27]. Hence, we evaluated the expression level of Iba-1, a classical marker of microglia, in the hippocampus of the rats by immunofluorescence. Consistent with the results of behavioral experiments, CMS significantly increased the protein level of Iba-1 in the hippocampus (Fig. 2A; *p* < 0.001). In addition, the microglia in the dentate gyrus area of the rats exposed to CMS showed amoeboid features in morphology, such as synaptic retraction as well as enlarged cell bodies (Fig. 2B), which is a typical activation pattern of microglia. Notably, the expression level of Iba-1 significantly fell back after chronic treatment of clomipramine in the hippocampus of the CMS-treated rats.(Fig. 2A, B; *p* < 0.05). Furthermore, qPCR revealed that the mRNA levels of a series of pro-inflammatory cytokines significantly increased (*p* < 0.001, *p* < 0.01, *p* < 0.01, respectively) and chronic clomipramine treatment prevented the increase in these mRNA levels in hippocampus caused by CMS (Fig. 2C–E; *p* < 0.05, *p* < 0.01, *p* < 0.01, respectively). Meanwhile, chronic clomipramine treatment increased the mRNA levels of anti-inflammatory cytokines (namely IL-4 and TGF-β) (Fig. 2F, H; *p* < 0.01, *p* < 0.01, respectively) when compared to the rats treated with saline.

### Chronic clomipramine treatment reverses CMS-induced increases in hippocampal IL-1β and NLRP3 inflammasome

**Fig. 3:**
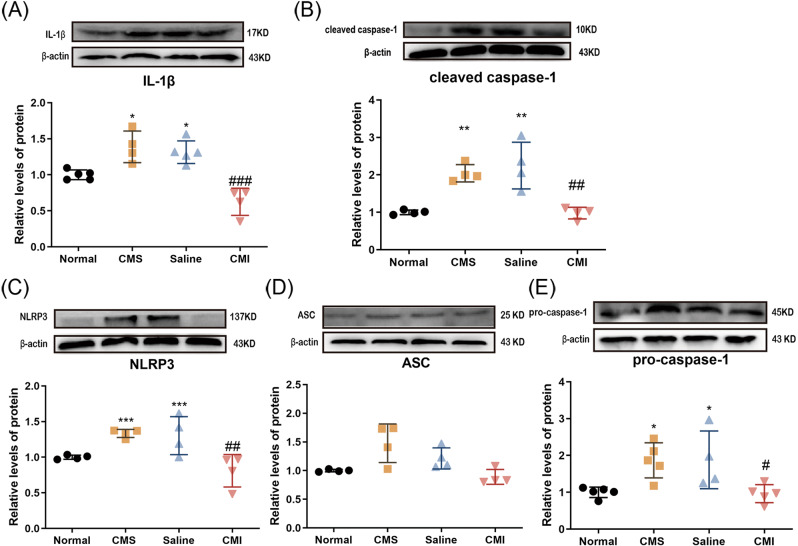
The effect of chronic clomipramine treatment on the hippocampal NLRP3 inflammasome level in the CMS-treated rats. The protein levels of **A** IL-1β, **B** cleaved caspase-1, **C** NLRP3, **D** ASC, and **E** pro-caspase-1 (*n* = 4/group) were analyzed by western blot. All data are expressed as the mean ± SD. ##*p* < 0.01, ###*p* < 0.001 compared to saline-treated rats. *p < 0.05, **p <0.01, ***p < 0.001 compared to non-stressed control rats.

Our previous work showed that IL-1β plays a role in stress-related depressive-like behaviors [26]. In the CNS, the NLRP3 inflammasome, which consists of NLRP3, caspase-1, and ASC, acts to cleave the precursor of IL-1β and IL-18 (two kinds of classical pro-inflammation cytokines) into active forms [28]. We found that the protein levels of IL-1β and its convertase, namely cleaved caspase-1, were both increased in the hippocampus of rats exposed to CMS (Fig. 3A, B; *p* < 0.05, *p* < 0.01, respectively). Likewise, the components of the NLRP3 inflammasome (Fig. 3C, E) rose notably in protein levels in CMS-treated rats (*p* < 0.001, *p* < 0.05, respectively). As expected, the protein levels of cleaved caspase-1 and IL-1β were normalized after chronic clomipramine treatment (Fig. 3A, B; *p* < 0.001, *p* < 0.01, respectively). Also, the increased NLRP3 and caspase-1 expression levels induced by CMS were significantly inhibited by chronic clomipramine treatment (Fig. 3C, E; *p* < 0.01, *p* < 0.05, respectively).

### Effects of CMS and chronic clomipramine treatment on hippocampal volume

**Fig. 4:**
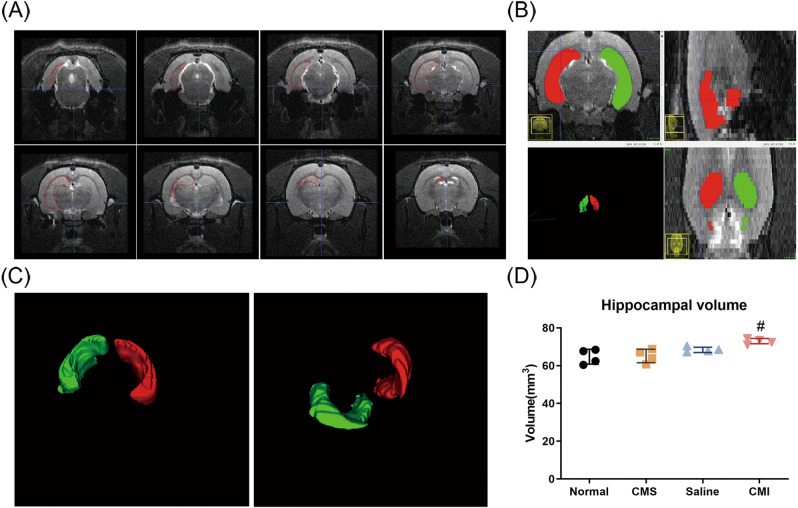
Effects of CMS and clomipramine treatment on the volume of the hippocampus. **A** Representative T2-weighted MRI image of rats. The area outlined in red represents the hippocampus. **B** The distribution of the delineated hippocampus in three planes. Different colors represent the hippocampus on different sides. **C** The three-dimensional rendering of the delineated hippocampus. Different colors represent the hippocampus on different sides. **D** MRI analysis of the rats’ hippocampi (*n* = 4/group). All data are expressed as the mean ± SD. #*p* < 0.05, compared to saline-treated rats.

We performed MRI scans on all four groups and manually delineated the hippocampus using ITK-snap. Then the hippocampal volumes were calculated and normalized (Fig. 4A–C). Surprisingly, we found that CMS fails to affect the hippocampal volume of the rats. On the contrary, however, the hippocampal volume of the rats treated with clomipramine was significantly higher than that of the rats treated with saline (Fig. 4D; *p* < 0.05).

## Discussion

Chronic mild stress is a reliable and effective paradigm to induce depression model in rodents. The consequences of unpredictable stress include a series of behavioral and physiological changes [29]. Here, we discovered that the rats under chronically stress status exhibited depressive-like behavioral outcomes and inflammatory changes, characterized by the elevated levels of NLRP3 inflammasome and pro-inflammatory cytokines, in the hippocampus. It has been found that the pro-inflammatory cytokines (mainly TNF-α and IL-1β) got a rise in the CNS of depressed subjects [30]. In addition, increased expression levels of NLRP3 inflammasome and pro-inflammatory cytokines have also been found [31–33], which is further confirmed by our results. As everyone knows, IL-1β is primarily processed by the NLRP3 inflammasome to its functional form in the CNS. NLRP3 (a kind of cytosolic sensor molecule), caspase-1 (a kind of effector molecule), and ASC (a kind of adaptor protein) constitute the NLRP3 inflammasome [34]. The present sites of NLRP3 inflammasome in the CNS are microglia and astrocytes [35]. Nevertheless, it has been found that in primary human nerve cells, NLRP3 and ASC mRNA could be detected in microglia, but less in astrocytes and neurons [36]. In addition, co-expression of NLRP3 and Iba-1, which suggested NLRP3 presented in the activated microglia, was observed in the prefrontal cortex of rats exposed to CMS [37]. All of these suggest that microglia might be the primary cell type where the NLRP3 inflammasome assembles and functions.

Microglia are thought to be the primary immune effector cells of the CNS. They secrete immune mediators to coordinate neuroinflammation rapidly as a response to the changes in the CNS microenvironment [38]. It has been found that stress can stimulate the hypothalamic–pituitary–adrenal axis, and as a result, circulating corticosterone levels increased significantly [39]. So the first areas to be affected are more likely to be areas with higher glucocorticoid receptor expression, and the hippocampus is an area like this [40]. Indeed, studies have suggested that stress can cause depression-related deficits in learning and memory [41–43], which is what the hippocampus is responsible for. So, it is not surprising that the microglia in the hippocampus are affected, especially since the microglia themselves express both mineralocorticoid and glucocorticoid receptors [44]. Our results showed that after CMS, increased hippocampal Iba-1 signal detected by immunofluorescence was found, which confirmed that microglia in the hippocampus of the rats that underwent stress were widely activated. As a result, significant increases in the hippocampal mRNA levels of pro-inflammatory mediators (namely IL-1β, IL-18, and IL-6) of the CMS-treated rats, accompanied by elevated expression levels of NLRP3 inflammasome, including its activated form, and its production IL-1β. Taken together, our results and previous studies confirmed that chronic stress could activate the microglia in the hippocampus, and further the NLRP3 inflammasome expression and activation increased resulting in elevated pro-inflammatory cytokines and ultimately leading to depression.

Since neuroinflammation is essential to the pathological process of depression, inhibition of neuroinflammation could contribute to the remission of depression. It has been found in many studies that fluoxetine, the classical antidepressant, and certain molecular extractions from traditional Chinese herbs (e.g., Icariin, L-menthone, etc.) can suppress the NLRP3 inflammasome level as well as its activation and play an antidepressant role in the rodents under chronic stress status [45, 46]. The antidepressant clomipramine is extensively used for psychiatric disorders treatment, including some anxiety disorders and depression [47]. Moreover, we found previously that clomipramine ameliorates several changes in behavior and molecular level in LPS-treated mice [16]. Besides, we found that the antidepressant effect of clomipramine is related to glial cell line-derived neurotrophic factor as well as glial fibrillary associated protein (GFAP) in rats exposed to CMS [15, 48]. Nevertheless, it is remaining unclear whether the mechanism by which clomipramine improves depression-related behaviors in CMS-treated rats is related to the improvement of neuroinflammation. In the present study, we assessed whether clomipramine exerted its antidepressant effects by suppressing neuroinflammation in rats exposed to CMS. First, not surprisingly, chronic clomipramine administration significantly improved CMS-induced depressive-like behavior. Based on our western blot and quantitative PCR results, chronic clomipramine administration completely inhibited the hippocampal expression of Iba-1, the microglial marker, together with NLRP3 inflammasome as well as pro-inflammatory cytokines. Moreover, we observed that clomipramine increased anti-inflammatory factors in the hippocampus of the clomipramine treatment group. In fact, in an acute depression model, we also found that clomipramine can regulate NLRP3 inflammasome and pro-inflammatory processes caused by LPS administration leading to the improvement of depressive-like behaviors [11]. All these results indicated that chronic clomipramine treatment ameliorated depressive-like behavior related to CMS by not only reducing pro-inflammatory contents but also promoting the anti-inflammatory process.

The detection of hippocampal volume through MRI scans has now become an important method for depression research. A number of clinical as well as preclinical studies have suggested that the pathology of depression is connected with decreased neurogenesis and hippocampal atrophy [49, 50]. Nevertheless, the conclusion about the changes in hippocampal volume related to depression is kind of controversial. While a study claimed that chronic stress or corticosterone administration had a negative effect on the hippocampal volume in rodents [51], still others have found the contrary results [52, 53], which are much more similar to our results. The inconsistencies of these conclusions may be partially due to the differences in hippocampal substructure segmentation. More advanced hippocampal substructure segmentation may help us find out more subtle volume changes in depression [54]. Besides, the changes in the hippocampal volume seem to be related to the duration of illness. A meta-analysis found that patients with a brief duration of illness (≤2.1 years) did not show significant hippocampal volume changes [55]. And in another clinical study, no variation in hippocampal volume was found in depressed patients, in which the authors indicated that the difference may be due to the patients’ age and gender [56]. Furthermore, from other preclinical studies, the number of animals in every cage and different stressors also seem to affect the results [19, 53]. Another possible cause for the different results is the small sample size, which is also a limitation of our research. Few studies, however, have reported the effect of clomipramine on hippocampal volume. Our study shows that chronic clomipramine treatment significantly increased the hippocampal volume for the first time, which may have something to do with the reverse of depressive-like behavior and neuroinflammation induced by CMS. Besides, it has been demonstrated that hippocampal cell proliferation, as well as neurogenesis, could be promoted by chronic clomipramine treatment [14, 57], which may account for the changes in hippocampal volume [58, 59]. On the other hand, clomipramine likely increased the rat’s hippocampal volume by promoting gliosis, which could have something to do with the increase of hippocampal volume under pathological conditions [60, 61] since our previous work indicated that chronic clomipramine treatment increased GFAP level in the hippocampus of rats exposed to chronic unpredictable stress [48]. Furthermore, Peixoto-Santos et al. found that the extracellular matrix also plays an important role in the maintenance of hippocampal volume except for cellular constituents [62]. Unfortunately, the relationship between clomipramine and extracellular matrix remains unknown, which needs future works to illustrate. The internal relationship between chronic clomipramine treatment and hippocampal volume changes may have something to do with the NLRP3 inflammasome. On the one hand, the inhibition of the NLRP3 inflammasome is parallel with attenuated neuronal damages in experimental ischemic stroke [63]. In another study, it has been demonstrated that the inhibition of the NLRP3 inflammasome is related to enhanced neurogenesis in an LPS-induced model [64]. Combined with our results, chronic clomipramine treatment may increase the hippocampal volume through the regulation of the NLRP3 inflammasome, which affects both neuronal damage and neurogenesis. Besides, gliosis and extracellular matrix may be affected by clomipramine to change the hippocampal volume. However, the exact mechanism remains to be further studied.

In conclusion, our findings indicate that under CMS status, the antidepressant clomipramine was capable of improving depressive- and anxiety-like behavior, neuroinflammation, as well as the expression of NLRP3 inflammasome. Among them, NLRP3 inflammation may play a particularly vital role. And chronic clomipramine treatment ameliorates these changes induced by CMS. Moreover, CMS-induced changes in hippocampal volume were not detected by MRI. The hippocampal volume of the clomipramine-treated group, however, was been found significantly increased in the rats exposed to CMS compared to that of the saline-treated group. Meanwhile, our study has many limitations. we did not take other methods like measuring in the brain sections to obtain hippocampal volume except MRI, which may lead to a slight bias in our results. Further research is needed to explore the potential mechanism and validate our conclusion.
